# Molecular and physicochemical arrangement of chitosan–ibuprofen matrices for topical drug delivery on skin: preparation impact

**DOI:** 10.1039/d6ra00296j

**Published:** 2026-03-19

**Authors:** Barbara Gieroba, Vladyslav Vivcharenko, Grzegorz Kalisz, Paulina Kazimierczak, Olena Mozgova, Maryna Khalavka, Liudmyla Nosach, Izabela S. Pięta, Robert Nowakowski, Agata Przekora, Anna Sroka-Bartnicka

**Affiliations:** a Independent Unit of Spectroscopy and Chemical Imaging, Medical University of Lublin Chodźki 4a Street 20-093 Lublin Poland barbara.gieroba-siwiec@umlub.edu.pl anna.sroka-bartnicka@umlub.edu.pl +48-81448-7320 +48-81448-7322; b Department of Tissue Engineering and Regenerative Medicine, Medical University of Lublin Chodźki 1 Street 20-093 Lublin Poland; c Department of Bioanalytics, Medical University of Lublin Jaczewskiego 8b Street 20-090 Lublin Poland; d Institute of Physical Chemistry Polish Academy of Sciences Kasprzaka 44/52 Street 01-224 Warsaw Poland; e Independent Unit of Translational Biomedical Research, Medical University of Lublin Chodźki 4a Street 20-093 Lublin Poland; f Department of Amorphous and Structurally Ordered Oxides, Chuiko Institute of Surface Chemistry, NAS of Ukraine 17 General Naumov St. 03164 Kyiv Ukraine

## Abstract

Recent advances in modern medicine emphasize patient-centric and personalized therapeutic strategies, particularly for chronic and regenerative applications. Among emerging biomaterials, chitosan (CS) has gained considerable attention due to its biocompatibility, biodegradability, and antimicrobial properties, while its molecular weight strongly influences structural organization and interactions with active compounds. In this study, chitosan-based matrices—2% (w/v) low-molecular-weight CS, 4% (w/v) low-molecular-weight CS, and 2% (w/v) medium-molecular-weight CS—were developed and enriched with ibuprofen (IBU), a widely used non-steroidal anti-inflammatory drug, to improve topical delivery and reduce systemic side effects. The physicochemical properties of gelled thin films were investigated with emphasis on molecular arrangement, surface characteristics, and drug release behavior in phosphate-buffered saline. ATR-FTIR spectroscopy, contact angle measurements, and atomic force microscopy (AFM) were employed to evaluate structural and functional changes induced by IBU incorporation. Medium-molecular-weight CS exhibited lower water contact angles (≈69–73°) and higher surface free energy (≈41–44 mN m^−1^) compared to low-molecular-weight CS, while IBU loading did not significantly alter wettability. AFM analysis revealed drug-induced surface roughness changes, with Ra increasing from 23.9 nm (2CS_M) to 29.4 nm after IBU loading and further to 35.5 nm following release. ATR-FTIR spectra confirmed preservation of characteristic chitosan amide I and II bands (∼1645 and ∼1556 cm^−1^), with spectral changes in the 1450–1700 cm^−1^ region indicating interactions between IBU and CS functional groups. Among the investigated systems, 2% (w/v) medium-molecular-weight chitosan demonstrated the most favorable sustained IBU release (∼50% after 48 h), highlighting its potential for dermal drug delivery and personalized therapeutic applications.

## Abstract 2

Recent advances in modern medicine emphasize patient-centric and personalized therapeutic strategies, particularly for chronic and regenerative applications.

## Graphical abstract

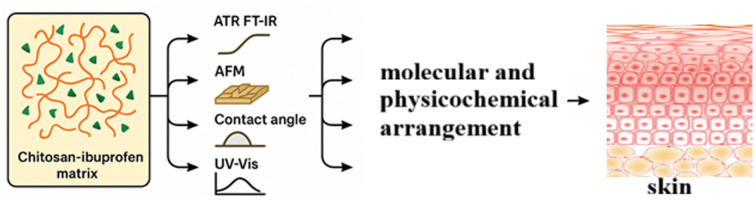

## 1. Introduction

Modern medicine and pharmacy have evolved in recent years aiming at different health and environmental issues. A highly patient-oriented approach tailors medical treatment solutions based on individual needs, states, and characteristics.^1^ It involves customization of formulation, and dosage, leading to a higher success rate of curing diseases or more effective therapy improving patients' life quality.^1^ The premise of integrating individual information from genome and phenotype leads to a proactive, preventive treatment model with reduced adverse reactions which may be implemented in chronic conditions or regenerative medicine.^2,3^ The term personalized medicine in this context usually relates to the use of stem cells in injuries, diabetes, heart disease, *etc.* However, the development of bioartificial organs or specific matrices for regeneration can also be considered here. Personalized, shaped treatment options can be developed based on predicted material features, to ensure maximum biocompatibility and bioactivity, possibly by applying naturally derived compounds. One of the promising natural polymers is chitosan (CS), poly[β-(1 → 4)-linked-2-amino-2-deoxy-d-glucose], obtained by alkaline deacetylation of chitin. It consists of d-glucosamine and *N*-acetyl-d-glucosamine units, linked by β-1,4 glycosidic bonds, and can be found in exoskeletons of crustaceans.^4,5^ It leads to variations in structure, size, and monomer distribution and allows the acquisition of different engineered products by modifying production methodology, or using various techniques (*e.g.* microwaves).^6,7^ CS is biocompatible, biodegradable, and non-toxic, it has antimicrobial properties and can form films, hydrogels, and nanoparticles. CS can be produced from krill exocuticles through a multistage process, involving degreasing, demineralization, and deproteinization, resulting in a chitin content of 92.8% and a deacetylation degree of 74–88% The resulting CS can be classified into high (HMW CS, >700 kDa), medium (MMW CS, 150–700 kDa), and low molecular weight CS (LMW CS, less than 150 kDa).^8,9^ It influences the properties of chitosan and its derivatives, like hydrophilicity, crystallinity, tensile strength, elastic modulus, moisture content, degradation, and finally protein adsorption and cell response.^10–12^ LMW CS was even associated with better solubility and antimicrobial properties but lower permeability due to a less densely packed polymer.^13,14^ This structural property also influences substances' release rates, in materials intended as a drug delivery system.^15^ When CS is exposed to an acidic environment, the amino groups in the polymer chains protonate, creating NH_3_^+^ moieties in its backbone.^16^ It allows the creation of a cationic matrix, interacting with different types of molecules, charge density, crystallinity and even propensity to enzymatic degradation.^17–19^ This natural, highly biocompatible polymer thus has drug delivery capabilities, allowing chemical stability, and was explored as carrier material. The cationic character of cross-linked chitosan improves applicability, by better efficiency of bioadhesion and absorption, which is a rare characteristic for natural polysaccharides.^20^

Different authors have proposed various methods of obtaining chitosan, by chemical (sodium hydroxide) and physical treatment (high temperature, pressure, irradiation).^21–23^ The resulting products differed in the degree of acetylation, molecular weight (MW), or crystallinity.^23^ Further, the employment of diverse substrates, regarding the above-mentioned properties, will affect engineered products, tissue scaffolds, wound dressings, or drug carriers according to the chosen methodology of preparation.^24–26^ For example, a more regular arrangement allows for strong intermolecular interactions, such as hydrogen bonding between amine and hydroxyl groups of adjacent chains.^27^ Higher molecular weight CS tends to have higher crystallinity through longer polymer chains, which can be more easily aligned, with amino groups forming hydrogen bonds. The opposite, random arrangement can lead to more flexible and accessible matrices to solvents and other molecules.^28^ Rapid drying of CS or high-temperature treatments can increase crystallinity by promoting chain alignment. Depending on chain interaction, cross-linking can also influence structure order or interactions based on hydrogen bonding, van der Waals forces, and electrostatic interactions. Those qualify CS to interact with biologically active molecules, *e.g.* antibiotics, vitamins, or non-steroid anti-inflammatory drugs (NSAIDs).^29–31^ It was repeatedly reported in the literature that CS and its derivatives have good antimicrobial activity, biocompatibility, and biodegradability and it was suitable for potential medical applications in gastrology, gynecology, surgery, dermatology, and many others.^32–35^ Antimicrobial and antifungal properties can be supported by anti-inflammatory compounds, *e.g.* for injury treatment with NSAIDs-enriched CS films for local analgesic effect.^36^ Ibuprofen is a chiral NSAID with anti-inflammatory, analgesic, and antipyretic properties, which can be used for pain palliation associated with inflammation, including rheumatoid arthritis and postoperative analgesia. Ibuprofen (IBU) is a non-selective reversible inhibitor of the cyclo-oxygenase isozymes COX-1 and COX-2, which are responsible for the conversion of arachidonic acid into prostaglandins including thromboxane and prostacyclin 37,38. The administration of IBU is mostly by oral route every 6 hours with tablets, capsules, or solutions. The therapeutic dosage for an adult is around 20–30 mg.^37^ However, due to the drug's extensive first-pass metabolism and low solubility leading to poor absorption, the administered dose is typically more than ten times this amount, which leads to adverse effects. Gastritis, dyspepsia, epigastric pain, heartburn, and peptic ulcer/gastrointestinal bleeding can be associated with major IBU side effects, which can be avoided by implementing it in a topical, degradable formulation.

In this work, CS matrices prepared with various concentrations and molecular weights, enriched with ibuprofen were investigated as a promising material for topical application on the skin. Physicochemical features and molecular arrangements of gelled thin films with or without IBU were evaluated. Interfacing surface properties were investigated by combined analytical techniques: Fourier transform infrared spectroscopy in attenuated total reflection mode (ATR FT-IR), contact angle measurements (wettability), and atomic force microscopy (AFM). Characteristics related to CS films were assessed aimed at changes imposed by IBU enrichment and drug release in phosphate-buffered saline (PBS).

## 2. Materials and methods

### 2.1. Samples preparation

Chitosan matrices were produced using 2% (w/v) chitosan with low molecular weight (59.8 kDa estimated based on the chitosan solution viscosity measurement, according to the protocol described earlier,^39^ Sigma-Aldrich Chemicals, Poland; labelled as 2CS_L), 4% (w/v) chitosan with low molecular weight (59.8 kDa, Sigma-Aldrich Chemicals, Poland; labelled as 4CS_L), and 2% (w/v) chitosan with medium molecular weight (217 kDa estimated based on the chitosan solution viscosity measurement, provided by the National Marine Fisheries Research Institute, Gdynia, Poland; labelled as 2CS_M). The ibuprofen-loaded chitosan matrices were fabricated by adding ibuprofen (Sigma-Aldrich Chemicals, Warsaw, Poland) to each of the above-mentioned chitosan matrices (samples labelled as 2CS_L/IBU, 4CS_L/IBU and 2CS_M/IBU) at the production stage.

To produce the chitosan matrices, appropriate amounts of chitosan were dissolved in 600 µL of 1% (v/v) acetic acid (CH_3_COOH, Avantor Performance Materials, Gliwice, Poland) and then 8 µL of 30 mg per mL ibuprofen solution prepared in ethanol were added. For uniform distribution of ibuprofen within the matrices, the dissolved chitosan was mixed with the ibuprofen solution using a magnetic stirrer. Subsequently, the resultant gels (containing 240 µg dose of ibuprofen) were spread in a thin layer onto 15 mm × 15 mm glass coverslips, forming an individual sample with area of 2.25 cm^2^. Next, samples were soaked in 1% (w/v) sodium hydroxide (NaOH, Avantor Performance Materials, Gliwice, Poland) for 5 minutes, rinsed with deionized water, and allowed to air dry. Prior to testing, the dried matrices were fully detached from the glass coverslips. The thickness of the dried samples was measured with an electronic micrometer (Schut Geometrical Metrology, Groningen, The Netherlands), which has an accuracy of 0.001 mm. The resulting chitosan matrices had a thickness of 90 µm ± 9.6 µm.

### 2.2. Evaluation of drug release

To assess ibuprofen release from the ibuprofen-loaded matrices (2CS_L/IBU, 4CS_L/IBU, 2CS_M/IBU), the samples were placed in 10 mL of phosphate-buffered saline (PBS) solution (pH 7.4, Sigma Aldrich-Chemicals, Poland) and incubated at 37 °C. Although 37 °C represents core body temperature, it is frequently employed in release studies to allow comparison with existing literature and to evaluate drug diffusion behavior under well-controlled conditions. Moreover, skin temperature at an inflammation site typically increases by approximately 1–2 °C compared to surrounding healthy skin.^40^ In turn, PBS was selected as the release medium due to its defined pH, ionic strength, and buffering capacity, which help maintain stable experimental conditions and ensure the chemical stability of ibuprofen throughout the test. Matrices without ibuprofen were placed in PBS in an analogous manner and served as control samples during physicochemical tests. At determined time points, 0.5 mL samples were taken to measure ibuprofen concentration, and fresh PBS was added to maintain the original volume. The concentration of ibuprofen was determined by measuring absorbance at 225 nm using a UV-spectrophotometer (Genesys 6 UV-Vis, Thermo Fisher Scientific, Waltham, MA, USA). A calibration curve was generated using known ibuprofen concentrations in PBS, ranging from 12.5 to 400 µg mL^−1^. The cumulative ibuprofen release profile from the chitosan matrices was depicted as the percentage of drug released over time. Results are reported as mean values ± standard deviation (*n* ≥ 3). Statistical analysis was conducted using one-way ANOVA followed by Tukey's test, with significance set at *p* < 0.05 (GraphPad Prism 8.0.0 Software, GraphPad Software Inc., La Jolla, CA, USA).

### 2.3. Kinetic modeling of ibuprofen release

The kinetics of ibuprofen release from chitosan-based matrices were analyzed to elucidate the dominant release mechanism and to assess the influence of chitosan molecular weight and concentration. Release experiments were conducted in phosphate-buffered saline (PBS), and the cumulative amount of ibuprofen released (*M*_*t*_) was expressed as a percentage of the total drug content (*M*_∞_). The experimental release data were fitted to commonly used Korsmeyer–Peppas kinetic models for polymer-based drug delivery systems. The model was applied to the initial stage of release (*M*_*t*_/*M*_∞_ < 0.6) and is given by:

1 *M*_*t*_/*M*_∞_ = *kt*^*n*^

where *k* is a kinetic constant incorporating structural and geometric characteristics of the matrix, and *n* is the release exponent indicative of the dominant release mechanism. Linear regression analysis was used to determine kinetic parameters and coefficients of determination (*R*^2^). Model applicability was evaluated based on goodness of fit and consistency with the physicochemical characteristics of the investigated systems.

For linear regression analysis, the equation was transformed into its logarithmic form:2
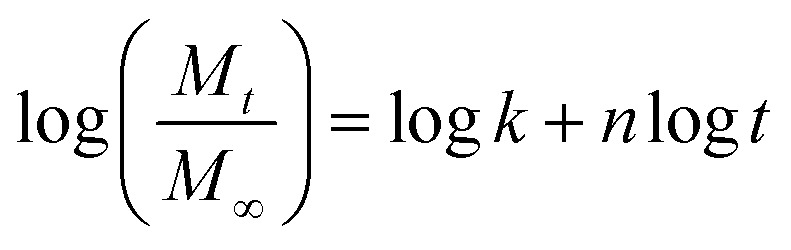
Thus, the slope of the linear fit corresponds to the release exponent (*n*), while the intercept corresponds to log *k*. The model was applied to the initial release stage (*M*_*t*_/*M*_∞_ < 0.6).^41^

### 2.4. ATR FT-IR spectroscopy

Infrared spectra were collected with the application of FT-IR Nicolet 6700 (Thermo Scientific, Waltham, MA, USA) spectrometer in the attenuated total reflection (ATR) mode. Spectra were recorded using Omnic 8 software from Thermo Fisher Scientific (Madison, WI, USA) in the 400–4000 cm^−1^ range. Each spectrum represented an average of 120 scans obtained at a resolution of 4 cm^−1^. The results are an average of 5 spectra for every sample after baseline correction measured at room temperature. Normalization was performed to the highest intensity band (∼1024 cm^−1^). To characterize the changes in the molecular structure in tested samples, second-order derivative spectra were calculated after processing (smoothing with nine points Savitzky–Golay algorithm). Spectral analysis was performed with Orange hyperspectral data processing suite, with Quasar software (ver. 1.5.0, Bioinformatics Lab, University of Ljubljana, Slovenia). After normalization intensity of absorbance for polar groups: amine C–N (∼1375 cm^−1^) and carboxyl COOH (∼660 cm^−1^), followed by *I*_1375_ : *I*_660_ ratio were calculated according to CS–IBU interaction described by Mahmoud *et al.*^42^

### 2.5. Evaluation of wettability

The static contact angle method determined the chitosan matrices' surface free energy using the DSA 30 Krüss goniometer (Krüss GmbH, Hamburg, Germany). Diiodomethane (Sigma Aldrich-Chemicals, Warsaw, Poland) and ultra-pure water were used for measurements as non-polar and polar liquids, respectively. The surface energies and their components (polar and dispersive values) were assessed based on an Owens, Wendt, Rabel, and Kaelble (OWRK) method. For contact angle measurements, at least three separate chitosan matrices were used and each of those was measured at least 6 times. Results are reported as mean values ± standard deviation (*n* ≥ 3). Statistical analysis was conducted using one-way ANOVA followed by Tukey's test, with significance set at *p* < 0.05 (GraphPad Prism 8.0.0 Software, GraphPad Software Inc., La Jolla, CA, USA).

### 2.6. Atomic force microscopy

The surface topography of the samples was imaged at a high resolution through atomic force microscopy (AFM, system Dimension Icon from Bruker). All images were collected in tapping mode using standard AFM probes (Bruker). For each sample, the microscopic investigations were repeated for different surface areas to gather statistical information. The presented images are representative of each sample.

Grain size values were determined from statistical analysis of grains identified in 800 nm × 800 nm AFM images (Gwyddion 2.59 software). The corresponding grain size distribution histograms and number of analyzed grains are presented in the SI.

All samples were tested before drug release test (prior to placing them in PBS) and after the test. For a more facilitated understanding of sample terminology, the summary is presented in Table 1.

**Table 1:** Abbreviations and description of investigated samples

	2% Chitosan low molecular weight	4% Chitosan low molecular weight	2% Chitosan medium molecular weight
Native	2CS_L	4CS_L	2CS_M
Native after release test	2CS_L_R	4CS_L_R	2CS_M_R
Ibuprofen-loaded	2CS_L/IBU	4CS_L/IBU	2CS_M/IBU
Ibuprofen-loaded after release test	2CS_L/IBU_R	4CS_L/IBU_R	2CS_M/IBU_R

## 3. Results

### 3.1. Drug release test

**Fig. 1:**
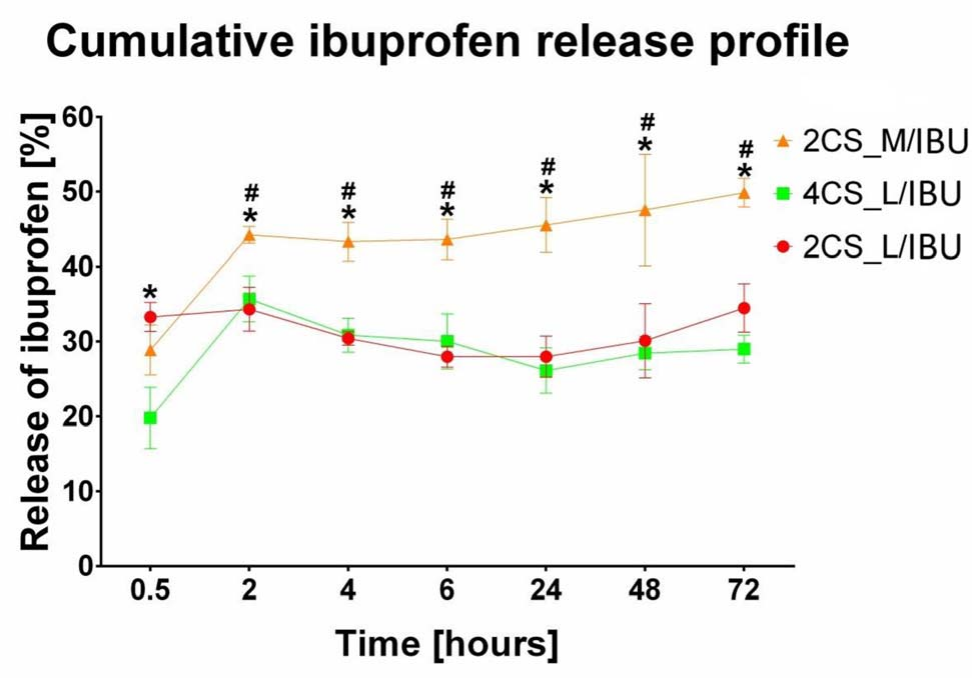
The cumulative ibuprofen release profile from ibuprofen-loaded chitosan samples, presented as the percentage of drug released over time (*statistically significant results compared to 4CS_L/IBU, ^#^statistically significant results compared to 2CS_L/IBU, *P* < 0.05, one-way ANOVA followed by Tukey's test).

The data obtained with drug release tests are presented in Fig. 1. The 2CS_L/IBU matrix showed a high initial burst release of the drug just after half an hour. This sample released approx. 35% of the ibuprofen and reached the plateau effect. The 4CS_L/IBU revealed a comparable release profile of the drug. It showed burst release of the drug followed by a plateau effect after 2 h of the experiment (approx. 30% of the drug was released). In contrast to the samples made of low molecular weight CS, the 2CS_M/IBU matrix showed initial burst release of the drug followed by slow and sustained ibuprofen release, reaching the plateau effect after 48 h (approx. 50% of the drug was released).

### 3.2. Ibuprofen release kinetics

The cumulative ibuprofen release profiles strongly depended on chitosan molecular weight and concentration. Low-molecular-weight matrices (2CS_L and 4CS_L) exhibited pronounced burst release followed by irregular, non-monotonic behavior, resulting in very poor correlation with the Korsmeyer–Peppas model (*R*^2^ = 0.054 and 0.083, respectively; Table 2). These systems therefore do not follow power-law kinetics and cannot be described by classical diffusion-based models.

**Table 2:** Kinetic parameters of ibuprofen release from chitosan-based matrices (Korsmeyer–Peppas model, *M*_*t*_/*M*_∞_ < 0.6)

Sample	Kinetic constant *k*	Release exponent *n*	*R* ^2^	Interpretation^44^
2CS_L/IBU	0.3187	−0.0117	0.0543	Model not applicable; burst-dominated release
4CS_L/IBU	0.2652	0.0292	0.0828	Non-Fickian behavior; irregular release
2CS_M/IBU	0.3592	0.0837	0.6987	Constrained, diffusion-limited release; highest kinetic regularity

In contrast, the 2CS_M matrix (Table 2) showed a gradual and sustained release profile, reaching ∼50% cumulative release after 72 h. Only this system demonstrated moderate linearity in the log–log domain (*R*^2^ = 0.6987) within the initial release region (*M*_*t*_/*M*_∞_ < 0.6). The calculated release exponent (*n* = 0.0837) is markedly below the theoretical Fickian value for slab geometry (*n* ≈ 0.5), indicating very weak time dependence and deviation from classical diffusion-controlled transport.^43^ The log–log representation (Fig. S1) illustrates that only the 2CS_M/IBU system exhibits moderate linearity, whereas low-molecular-weight matrices deviate markedly from power-law behavior.

The low *n* value suggests a constrained release regime characterized by limited chain mobility and restricted diffusion pathways within the denser medium-molecular-weight chitosan network. Overall, increasing chitosan molecular weight improves matrix integrity and kinetic regularity, leading to more sustained release, whereas low-molecular-weight systems are dominated by burst desorption and lack predictable kinetic behavior.

### 3.3. ATR FT-IR spectroscopy

**Fig. 2:**
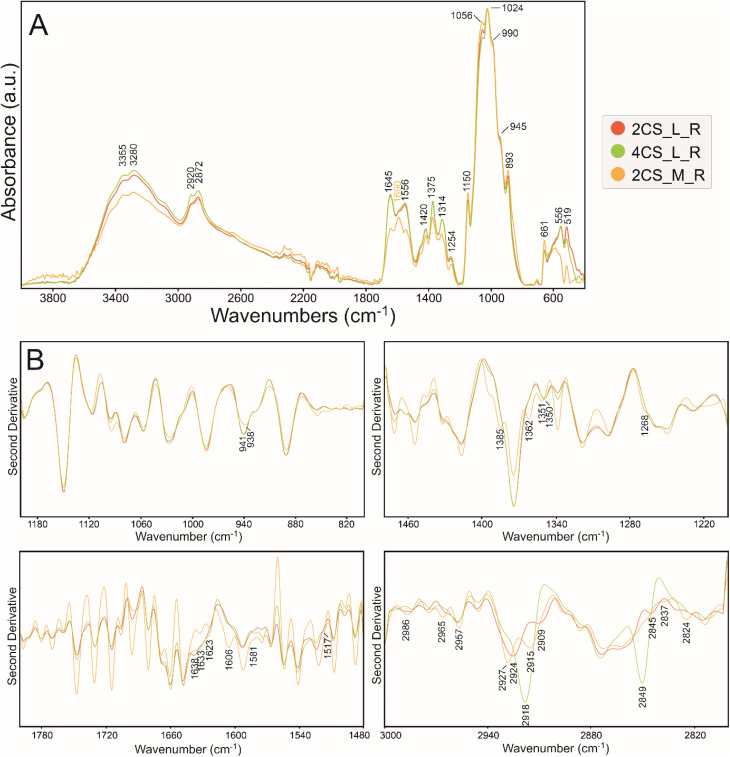
(A) ATR FT-IR spectra of control CS polymers spectra after release in PBS normalized to the highest intensity band at ∼1024 cm^−1^. (B) Their second derivative in ranges 800–1200 cm^−1^ (top left), 1200–1480 cm^−1^ (top right), 1480–1800 cm^−1^ (bottom left), and 2800–3000 cm^−1^ (bottom right).

**Fig. 3:**
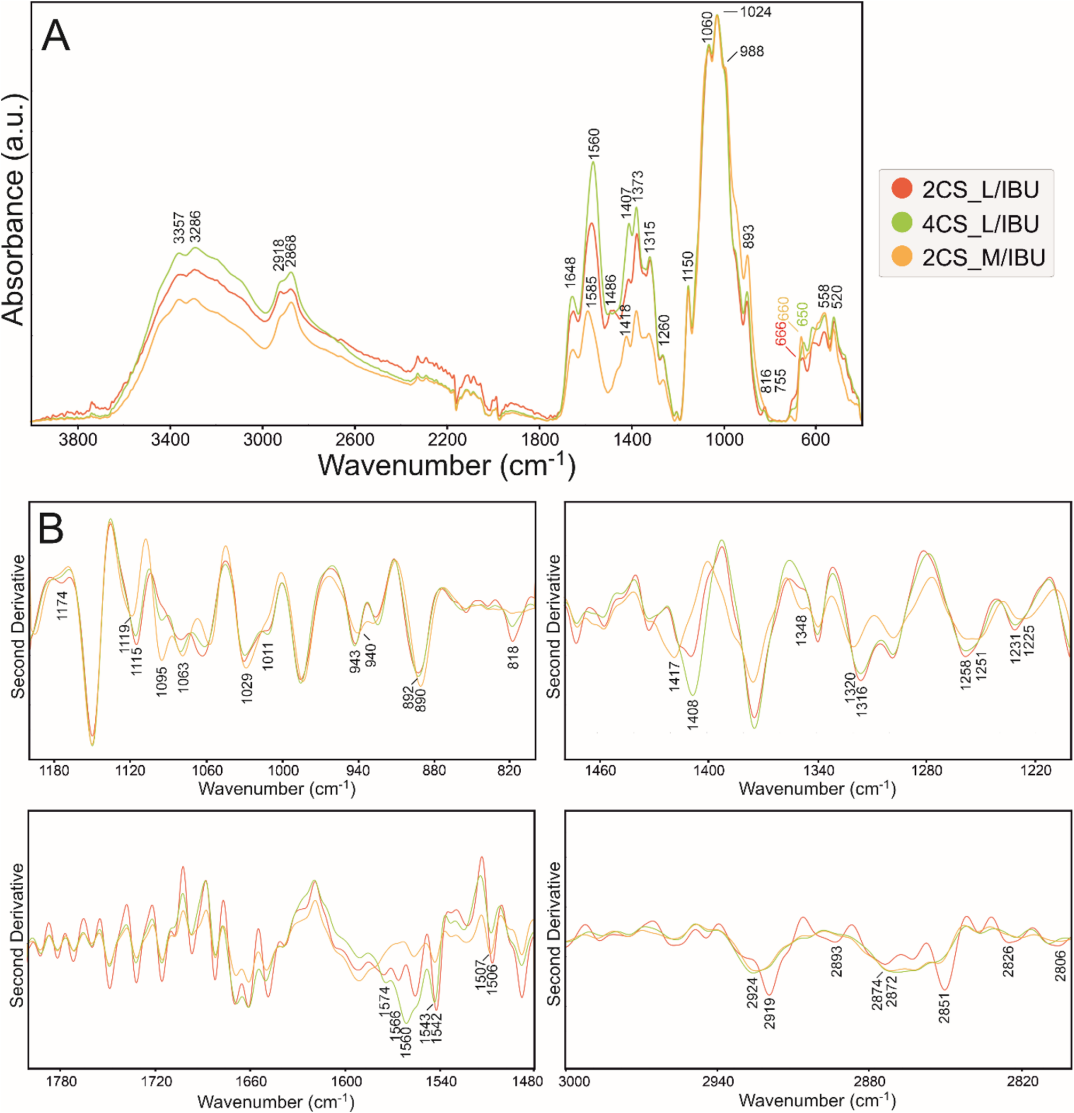
(A) ATR FT-IR spectra of ibuprofen-enriched polymers after release in PBS normalized to the highest intensity band at ∼1024 cm^−1^. (B) Second derivatives in ranges 800–1200 cm^−1^ (top left), 1200–1480 cm^−1^ (top right), 1480–1800 cm^−1^ (bottom left) and 2800–3000 cm^−1^ (bottom right).

ATR FT-IR spectra of matrices (2%, 4% low, and 2% medium MW CS), with ibuprofen and after release are presented in Fig. S2, 2A, 3A, and 4A accompanied with the second derivatives visualizations (Fig. 2B, 3B, and 4B). The 800–1200 cm^−1^ (fingerprint region), 1200–1480 cm^−1^ (amide III region), 1480–1800 cm^−1^ (amide I and II regions), and 2800–3000 cm^−1^ (associated with the presence of CH, CH_2_, and CH_3,_ bonds) spectral ranges were chosen for analysis.^45^ The most prominent bands in the vibrational spectra and the most important minima in the course of the second derivatives are assigned with wavenumbers on respective figures with color indication on most significant differences.

**Fig. 4:**
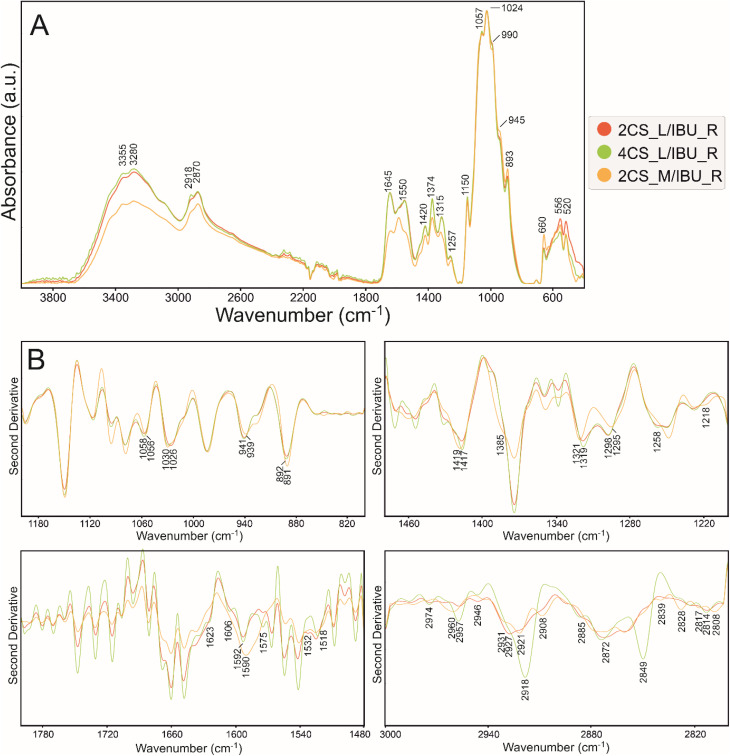
(A) ATR FT-IR spectra of ibuprofen-enriched polymers after release in PBS normalized to the highest intensity band at ∼1024 cm^−1^. (B) Second derivatives of spectra in ranges 800–1200 cm^−1^ (top left), 1200–1480 cm^−1^ (top right), 1480–1800 cm^−1^ (bottom left) and 2800–3000 cm^−1^ (bottom right).

The spectra, according to the literature, are typical for chitosan films and the assignments are summarized in Table S1 (ref. 46–49) in SI. In the spectra of the 2CS_M_R sample (Fig. 2A), the main change was detected in the range 1450–1700 cm^−1^ with an additional band at 1690 cm^−1^. Due to the different MW, 2CS_M_R differs in the intensity of absorption bands in the range 3000–3600 cm^−1^, which are attributed to hydrogen stretching.^50^ Both observations are connected with lower content of linked water and changes in amide region and C=O stretching.^51^ The carboxyl groups are more engaged in hydrogen bonding and more dense structure with local crystalline regions.^52^ It has been reported that chitosan with increasing MW showed the growing number of amino (1200–1500 cm^−1^) and a hydroxyl group (3550–2500 cm^−1^ broadband).^53^ This is also visible in the second derivative (Fig. 2B), where additional minima and shifts are present in the beta-sheet structure region indicating strong intermolecular bonding.^54^ Interestingly, the 4CS_L_R sample differs most in the 2800–2900 cm^−1^ range, assigned to symmetric and asymmetric stretching of C–H bonds in methyl and methylene groups.^55^ It can be a result of changes in the flexibility of the polymer chain in an aqueous environment followed by swelling of 4CS_L_R sample.

Then, the spectra of all variants were divided and presented into the IBU-loaded (Fig. 3), IBU-released (Fig. 4) to evaluate IBU-related changes in the structure of polymer.

IBU incorporation in 2CS_L, 4CS_L and 2CS_M matrices (Fig. 3A) resulted in changes in absorbance in ranges 1200–1700 and 2600–3600 cm^−1^, assigned to the secondary structure of a prepared polymer. Moreover, the presence of IBU carboxyl group involvement in bonding can be visible by shifts in the range of 650–666 cm^−1^ shift at 666, 660, and 650 cm^−1^ corresponding to C–H formatting vibrations of CH_2_=CH. The absorption bands in the 600–500 cm^−1^ part of the spectrum are of complex origin and correspond to contributions of bending and lattice modes.^56^ Ionic interactions or hydrogen bonding between the carboxylate group of IBU and hydroxyl and amine groups in matrices are visible (–NH) in the abovementioned spectral regions. 2CS_M/IBU can be characterized with the least significant spectral differences in investigated samples. The enrichment of IBU is less incorporated by ionic interactions and hydrogen bonding compared to low MW CS. In 4CS_L/IBU, higher intensity and a different course of the spectrum in the 1200–1717 cm^−1^ ranges were observed, which can be assigned to IBU absorption bands.^57^ Differences between the spectra in the 1800–1200 cm^−1^ range indicate a different binding mechanism of ibuprofen to studied chitosan matrices; there are also the lowest absorbance in 2CS_M/IBU sample (Fig. 3A). Bands corresponding to NH_2_ and OH stretching vibrations (amides region) were significantly shifted to lower wavenumbers in low MW specimens corresponding to the strong intermolecular hydrogen bonding between CS and IBU (Fig. 3A). It is suggested that the IBU molecule caused a change in the symmetry of chitosan due to the electrostatic interaction and hydrogen bonding between the carboxylic group of IBU and the protonated amino group of CS. This phenomenon may occur in low molecular weight samples. The second derivative in the range of 1200–800 cm^−1^ shows that the band at 818 cm^−1^ ascribed to C–O stretching appears in all samples, but is the most pronounced in the 2CS_L/IBU specimen (Fig. 3B).^58^ In this sample, there is also no band at 1095 cm^−1^ and a slight shift and different course of a band at 1063 cm^−1^ connected with C–O(H) stretching and anomeric structure of carbohydrates.^59^ In a 2CS_M/IBU sample, the disappearance of bands at 1010 and shift to 940 cm^−1^ is observed (C=O stretching and C–H deformational in β-glycosidic bonds, respectively).^60^ All these results prove the connection of polysaccharide units with ibuprofen. The most important difference is the shift of bands at 1408 cm^−1^ (due to methyl/methylene deformations and carboxyl group) and 1560 cm^−1^ (C–C stretching) in the 4CS_L/IBU sample.^61,62^ It may indicate a different mechanism of the ibuprofen–chitosan combination in all studied variants.

After IBU-release in PBS as presented in Fig. 4, an additional broad band in the second derivative of 1590 cm^−1^ (amide I range) appeared in 2CS_M/IBU_R, suggesting a change in the secondary structure. In 4CS_L/IBU_R at 2918 and 2849 cm^−1^ (Fig. 4A and B, CH_2_ and CH stretching vibrations, respectively), the absorbance is more pronounced.^55,63^ This may also represent a change in the conformation of the polysaccharide chains after PBS treatment, but in the surface groups, compared to medium MW CS. Sodium chloride in the PBS weakened the electrostatic interaction between amine ions of chitosan, presumably leading to a change in the conformation of the polymer chain and the appearance of C–H groups on the surface in Fig. 2 and 4.^64^ Subsequently, the intensity of absorbance ratio *I*_1375_ : *I*_660_ was determined (Table 3); these bands are assigned to polar amine C–N (∼1375 cm^−1^) and carboxyl COOH (∼660 cm^−1^) groups, reflecting CS–IBU interactions.

**Table 3:** Absorbance intensity of 1375 cm^−1^ and 660 cm^−1^ and their ratios (*I*_1375_ : *I*_660_) in the investigated samples

Sample	*I*_1375_	*I*_660_	Ratio
2CS_L/IBU	0.018295	0.051662	0.354131
2CS_L/R	0.036636	0.10368	0.353353
2CS_L/IBU_R	0.058131	0.136234	0.426702
4CS_L_IBU	0.064849	0.11144	0.581914
4CS_L_R	0.060403	0.137739	0.438529
4CS_IBU_R	0.029447	0.079075	0.372398
2CS_M/IBU	0.036235	0.12631	0.28687
2CS_M/R	0.014826	0.0609	0.243448
2CS_M/IBU_R	0.040945	0.140463	0.291502

Cationic polysaccharides, such as CS, consisting of ammonium groups and non-polar regions have been reported to provide hydrogen bonding capacity and high affinity for oppositely charged molecules, like ionized IBU, which in turn is a surface active compound able to adsorb onto polymers *via* hydrophobic and electrostatic bonds with their aromatic ring and hydrophilic carboxylic groups, respectively.^65^ It is worth noting that there might also be changes in the partial charge followed by a change in the total dipole moment. This kind of interaction is likely to take place through hydroxyl groups (OH⋯HO).^66^ These properties may influence the controlled release process of the drug (primarily due to the protonation of amino groups on the CS backbone at low pH, interacting with negatively charged ibuprofen IBU), highly improving biopharmaceutical applications.^65,67^ Analyzing the results presented in Table 2, the 4CS_L_IBU sample (0.581914) indicates a relatively strong interaction between the amine and carboxyl groups, suggesting enhanced binding or complex formation, while the 2CS_M/R sample has the lowest ratio (0.243448), suggesting weaker interactions in this case. The analysis reveals that the interactions between CS and IBU vary significantly based on formulation, with higher chitosan concentrations and the presence of IBU generally enhancing the interaction as reflected in both absorbance values and ratios. 4CS samples generally show higher absorbance intensity values and ratios compared to 2CS samples, indicating that increasing CS concentration may enhance interactions with IBU. Samples containing ibuprofen (like 2CS_L/IBU and 4CS_L_IBU) consistently show higher absorbance values at 1375 cm^−1^ compared to their counterparts without IBU, suggesting that amino groups interact with IBU.

### 3.4. Atomic force microscopy

**Fig. 5:**
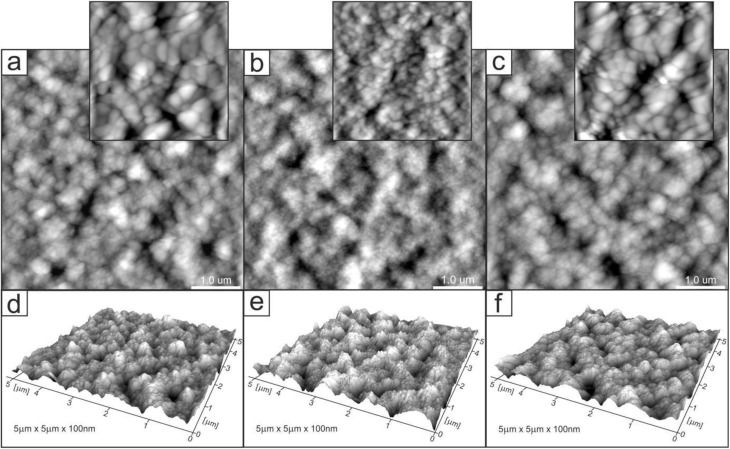
2D (a–c) and 3D (d–f) AFM images of 2CS_L_R (*R*_max_ = 134 nm) (a and d), 2CS_L/IBU (*R*_max_ = 132 nm) (b and e), 2CS_L/IBU_R (*R*_max_ = 110 nm) (c and f); zoom in (a) and (b) are 800 nm × 800 nm; 3D representations normalized to a height of 100 nm. Scanning area: 5 × 5 µm.

**Fig. 6:**
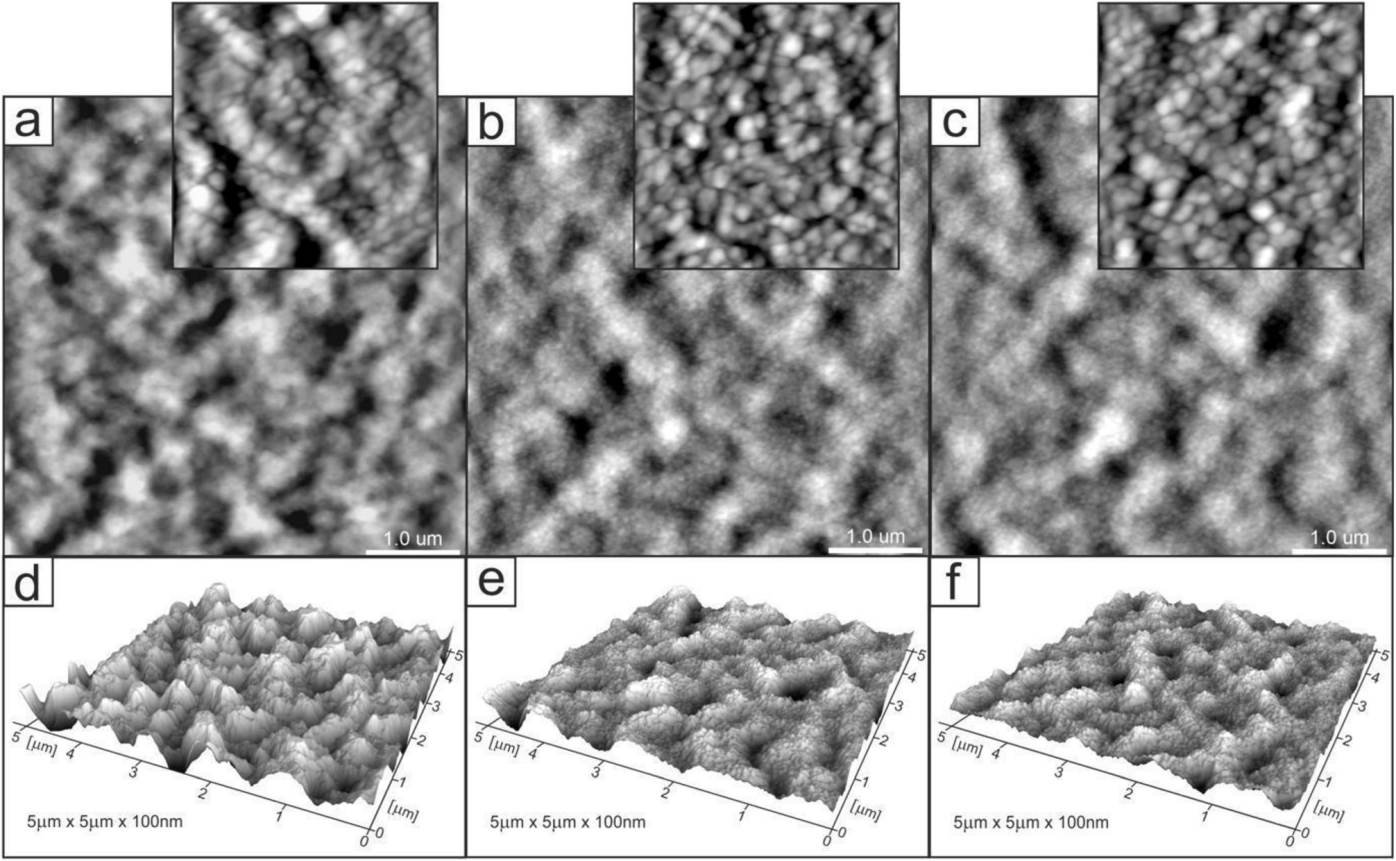
2D (a–c) and 3D (d–f) AFM images of 4CS_L_R (*R*_max_ = 180 nm) (a and d), 4CS_L/IBU (*R*_max_ = 106 nm) (b and e), 4CS_L/IBU_R (*R*_max_ = 102 nm) (c and f); zoom in (a) and (b) are 800 nm × 800 nm; 3D representations normalized to a height of 100 nm. Scanning area: 5 × 5 µm.

**Fig. 7:**
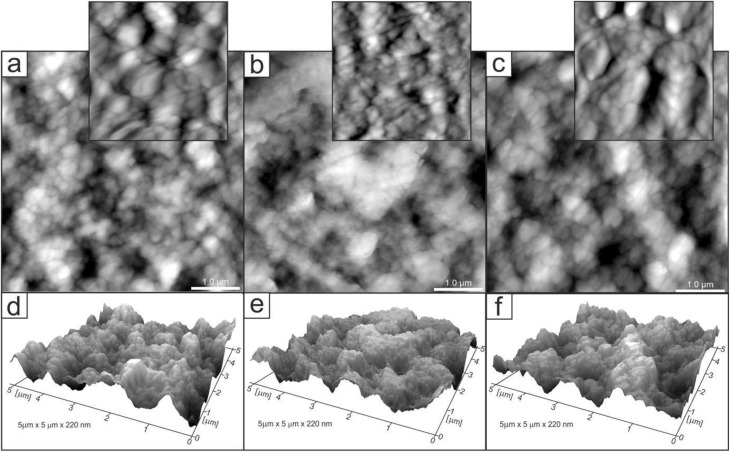
2D (a–c) and 3D (d–f) AFM images of 2CS_M_R (*R*_max_ = 177 nm) (a and d), 2CS_M/IBU (*R*_max_ = 216 nm) (b and e), 2CS_M/IBU_R (*R*_max_ = 285 nm) (c and f); zoom in (a) and (b) are 800 nm × 800 nm; 3D representations normalized to a height of 220 nm. Scanning area: 5 × 5 µm.

Changes in the topology of the chitosan layer induced by the introduction and release of ibuprofen. Fig. 5–7 show AFM images of representative layers: 2% chitosan low molecular weight, 4% chitosan low molecular weight and 2% chitosan medium molecular weight. For each sample, the imaging was performed for three layers at different stages of the experimental procedure: control chitosan layer and layers after introducing and releasing ibuprofen. In addition, exemplary profiles of selected lines are included in Fig. S3–S5 in SI. Generally, the surfaces of all layers were characterized by a grain structure. Locally, a tendency of grains to organize into longer linear structures could be observed. Significant observations concern discrete changes in quantitative properties. For all three types of the investigated samples, the layers with ibuprofen (images (b), and corresponding profiles) are characterized by significantly smaller grain sizes as compared to grains observed in control and drug-released layers. For example, in the case of sample 2CS_L (Fig. 5), the average grain size was 80 ± 28.1 nm for the control sample, 56 ± 17 nm for the ibuprofen-loaded sample, and after drug release a bimodal distribution was observed, with two grain populations of 90 ± 10 nm and 68 ± 22 nm. These two populations, correspond to the grains observed in the control and ibuprofen-loaded samples, respectively. Significantly smaller grains in the layer with a drug can be interpreted as a consequence of two different factors.

Firstly, the introduction of ibuprofen may cause the drug to be uniformly distributed in the polymer matrix leading to the drug grains being smaller than the polymer grains. Secondly, the presence of ibuprofen may generate less hydrophilicity of the layer surface. Consequently, observation of smaller grains can be expected in the case of AFM imaging in ambient conditions due to a thinner layer of adspecies (water) adsorbed on the layer surface from the air. Regardless of interpretation, the observed change confirms that the introduction of ibuprofen does not cause its aggregation in the layer leading to the formation of large clusters in the polymer matrix. In this case, we would observe inhomogeneities on the surface in the form of macroscopic regions with different chemical and physical properties. This is not the case. The change in the surface properties due to the introduction of ibuprofen while maintaining its homogeneity indicates uniform distribution of the drug.

Additional information is provided by comparing the surface roughness. The layer with ibuprofen, although it consists of the smallest grains, is characterized by higher roughness, which is confirmed by the surface development factor (the ratio of the real surface area to the projected area) and the *R*_a_ and *R*_q_ roughness parameters. For example, in the case of the sample discussed in detail (2CS_L), the parameters determined from the presented AFM images (Fig. 5) are as follows: 2.72%, 17.2 nm, 13.6 nm (control), 3.82%, 18.3 nm, 14.7 nm (with ibuprofen), 1.89%, 15.3 nm, 12.2 nm (after release). It is worth emphasizing that the control and drug-released layers are characterized by grains of similar size, which can indicate the complete release of the drug during the experimental procedure. However, the applied experimental cycle, namely the introduction and release of ibuprofen, leads to changes in the layer roughness. In the case of the discussed sample, the layer after the release of ibuprofen is less rough in comparison to the control layer. This fact can be associated with the presence of the drug in the layer and its interaction with the polymer leading to the reorganization of the carrier itself. Another comparison concerns the effect of chitosan size on the layer topology (2CS_L and 2CS_M samples, Fig. 6 and 7). Comparison of corresponding layers (control or with ibuprofen or after release) shows very similar grain sizes. The observation indicates that the size of grains as determined from AFM images does not depend on the molecular weight of chitosan in the studied range. However, the noted difference concerns the roughness of the layers. The roughness parameters determined from the AFM images for the 2CS_M sample are 3.7%, 23.9 nm, 19.0 nm (control), 5.34%, 29.4 nm, 23.4 nm (with ibuprofen), 4.71%, 35.5 nm, 28.5 nm (after release). Hence, the layers with larger polymers are more rough (note the different *z*-axis scale in 3D images and line profiles in Fig. S3–S5). The corresponding grain size distribution histograms and number of analyzed grains are presented in Fig. S6.

### 3.5. Wettability test

The wettability test revealed that hydrophilic surfaces characterized all chitosan matrices. However, the surface of the 2CS_M and 2CS_M/IBU showed significantly lower water contact angles than other samples (Table 4). Similarly, matrices made of medium molecular weight chitosan showed significantly lower diiodomethane contact angle compared to the samples based on the low molecular weight chitosan. It indicates that the molecular weight of the chitosan molecule directly affected the wettability of the resultant chitosan-based matrices. Consequently, surface free energy (SFE) calculated for the 2CS_M and 2CS_M/IBU was significantly higher than SFE values determined for other samples, confirming that these samples were more easily wetted. Considering obtained SFE values it may be observed that the addition of the ibuprofen to the chitosan matrices did not influence their wetting properties.

**Table 4:** Water and diiodomethane contact angle measurements along with calculated SFE for the tested samples^a^

Biomaterial sample	Average contact angle [°] ± standard deviation		SFE [mN m^−1^]
	Measuring liquid		
	Water	Diiodomethane	
2CS_L	84.55 ± 2.91^a,b,d^	55.47 ± 3.55^a,b^	34.91 ± 3.13^b,c^
2CS_L/IBU	81.79 ± 2.17^c^	55.02 ± 3.81^c^	36.1 ± 3.17^b,c^
4CS_L	89.75 ± 4.02^b^	52.43 ± 3.34	34.78 ± 2.91^b,c^
4CS_L/IBU	82.28 ± 3.36^a,c^	56.44 ± 2.74^a,c^	35.3 ± 2.92^b,c^
2CS_M	68.9 ± 6.37	50.68 ± 3.52	43.66 ± 5.35
2CS_M/IBU	72.75 ± 9.33	52.19 ± 2.25	41.17 ± 5.81

a Statistically significant results compared with ^a^4CS_L, ^b^2CS_M, ^c^2CS_M/IBU, ^d^2CS_L/IBU, where *p*-value <0.05; one-way ANOVA followed by Tukey's test, *n* ≥ 15.

## 4. Discussion

Modern medicine is characterized by its dynamic and interdisciplinary nature, continually advancing to address emerging health challenges and improve patient care. It needs a deep understanding of physicochemical material characteristics, to maximize the quality of products. Chitosan, as a derivative collected *e.g.* from natural krill chitin, is a sustainable source due to its abundance and rapid reproduction rates.^9^ Several physical chemistry techniques can be used to investigate the properties of produced materials, such as spectroscopy, spectrometry, microscopy, calorimetry, *etc.* However, considering natural polymers as a topical remedy, most surface-describing methods can be used. In this study, a combination of ATR FT-IR, AFM, and contact angle were applied to evaluate three preparation methodologies of CS thin films, as an ibuprofen-carrier.

The drug release tests provide critical insights into the behavior of chitosan matrices, particularly regarding the influence of molecular weight on the release kinetics and profile of ibuprofen. Importantly, maintaining a uniform thickness across all tested samples minimized geometric variability and ensured comparable experimental conditions in this study. Control of matrix thickness is essential because it directly affects the diffusion path length of the drug and, consequently, the release kinetics. However, topical patches may vary depending on formulation and therapeutic application. For instance many commercially available products based on drug-eluting polymer matrices have a thickness in the range of 50–7000 µm.^68,69^ The most important issue is finding a balance between drug release control and ease of administration and patient comfort.

Based on the obtained results, the burst release in LMW CS is likely attributed to their less dense structure and greater accessibility of the drug to the surrounding environment (the rapid dissolution of ibuprofen located near the surface of the matrices), which is common in drug delivery systems utilizing hydrophilic polymers. In contrast, the MMW CS matrix displayed a markedly different release profile. While it also exhibited an initial burst release, the subsequent release phase was notably slower and more sustained, with approximately 50% of the drug released after 48 hours. This sustained release can be attributed to the characteristics of medium molecular weight chitosan, which may provide a denser network that limits the diffusion of ibuprofen (formation of a more restrictive environment for the ibuprofen, leading to a prolonged release). The slower release rate indicates that the drug is not only embedded within the polymer matrix but is also subjected to a more complex release mechanism, potentially involving interactions between ibuprofen and the chitosan matrix that slow down drug diffusion. These findings underscore the importance of selecting appropriate chitosan molecular weights for specific drug delivery applications, particularly when a controlled release is desired. The ability to modulate the release rate through molecular weight variation provides a valuable tool for optimizing therapeutic outcomes in drug delivery systems.^70^ It should also be noted that the typical pH of healthy skin is approximately 4.0–6.0.^71^ Considering the pH-dependent solubility of chitosan, which is stable at physiological pH (7.4) but soluble in acidic media,^72^ it can be expected that the amount of drug released from chitosan matrices upon application to the skin will be higher than observed in experiment conducted at physiological pH.

To further elucidate the ibuprofen release mechanism, kinetic modeling was applied to the experimental data. The release profiles were evaluated using the Korsmeyer–Peppas models. Meaningful kinetic fitting was obtained only for the 2CS_M system, whereas low-molecular-weight chitosan matrices did not satisfy the assumptions of diffusion-controlled release models, likely due to rapid initial release and matrix reorganization.^73^ For the 2CS_M system, the Korsmeyer–Peppas model provided the best fit (*R*^2^ = 0.6987), with a low release exponent (*n* = 0.0837), indicating a diffusion-limited release mechanism strongly constrained by the polymer network.^74^ This behavior is consistent with spectroscopic and morphological observations, confirming that increased molecular weight leads to enhanced matrix rigidity, reduced chain mobility, and sustained drug release. In contrast, low-molecular-weight chitosan systems exhibited irregular release profiles that could not be adequately described by classical diffusion models, highlighting the dominant role of matrix restructuring and burst release effects.^75^

Physical chemistry techniques, such as spectroscopy and atomic force microscopy are currently more applied in biomedical analyses, for potential therapeutic agents. Their ability to identify specific functional groups or additives in the matrix is particularly important in formulations where chemical interactions can influence the drug's stability, release profile, or efficacy.^76^ Particularly a correlation of microscopic data and information of functional groups involved in interface and intramolecular interactions regarding visible surface are important in topical biomaterials. In this study ATR FT-IR spectroscopy has been used to determine the composition of the top surface layer of polymers enriched with IBU to compare how the substance release changes its surface. In the untreated sample subjected to drug release, the molecular structure of the matrix was not changed, with a significant difference of 1690 cm^−1^ band related to the N–H bending and C–N stretching and thus polymers' secondary structure of 2CS_M.^76^ Moreover, the enrichment with IBU leads to the reduction of absorbance in ranges 1200–1700 cm^−1^ and 2600–3400 cm^−1^ on the material surface, which is the result of stronger interaction by lowering the dipole moment, as indicated by Shirolkar *et al.*^77^ Moreover, the 2CS_M_R sample exhibits an additional band at 1690 cm^−1^ and notable shifts in the amide region (1450–1700 cm^−1^). This indicates a more pronounced alteration in the molecular interactions and hydrogen bonding in higher MW samples, likely due to increased amino group availability that enhances interaction with IBU. The resulting surface however is more rough for the 2CS_M sample, which suggests that the above-mentioned groups interacting with IBU are more internalized compared to low MW matrices. No characteristic absorption bands assigned to IBU were recorded in ATR spectra of the matrices surfaces.^78^ O'Callaghan *et al.* stated that low MW CS has greater surface charges than medium MW, possibly interacting with active compounds.^79,80^ In general, FTIR spectroscopy, which shows characteristic CS signals in CS/IBU without significant shifts or changes in intensity, indicates that ibuprofen loading does not alter the fundamental chemical structure of chitosan, as was proven by Olvera Rodríguez *et al.*^81^ It was proven in our study, where all spectra consistently display typical chitosan absorption bands, including the amide I (1645 cm^−1^) and amide II (1556 cm^−1^) regions, confirming the integrity of the chitosan matrix across different formulations and conditions. This suggests that the fundamental structural components of chitosan remain intact despite modifications. While structural integrity is maintained across formulations, the degree of interaction and resulting conformational changes highlight the importance of matrix composition in designing effective drug delivery systems. However, the introduction and release of IBU have significant effects on the topology of CS layers, as illustrated by the AFM images and profiles. One of the most notable findings is the reduction in grain size observed in layers with IBU. For instance, in the 2CS_L sample, the average grain size decreased from 90 nm in the control layer to 52 nm when IBU was introduced, before increasing again to 83 nm after drug release. This size reduction can be attributed to two primary factors. First, the uniform distribution of IBU within the CS matrix likely results in smaller drug grains compared to the polymer grains. Second, the presence of IBU may reduce the hydrophilicity of the layer, leading to a thinner water adsorption layer, which further influences the observed grain sizes. In order to verify the second hypothesis, subsequently wettability measurements were performed. Importantly, the smaller grain sizes during drug loading indicate that IBU does not aggregate within the matrix, as any significant clustering would have produced inhomogeneous surface features. Instead, the observed uniformity suggests that IBU is well-dispersed within the CS, contributing to a consistent surface morphology. This points to the formation of a homogenous structure with a well-proportioned network structure, dense architecture, and evenly distributed grains.^82^ Furthermore, the introduction of IBU correlates with an increase in roughness parameters, even though the grain size is smaller. For the 2CS_L sample, the surface development factor increased from 2.72% in the control to 3.82% with ibuprofen, reflecting a higher surface area due to the presence of smaller grains. This increase in roughness could be associated with the structural reorganization of the CS layer caused by the drug's interaction with the polymer matrix. Therefore, ATR FT-IR (also evidenced by the intensity of absorbance ratio *I*_1375_ : *I*_660_) and AFM studies prove electrostatic attraction between the protonated d-glucosamine monomeric unit of CS and the negative carboxylate ion of IBU significantly contributes to the polymer–drug interactions.^65^ Furthermore, hydrogen bonding between amine and hydroxyl groups in the CS and IBU molecules (carbonyl groups and oxygen atoms in the heterocyclic ring) presumably also participate in the binding mechanism. Besides, IBU may also form hydrogen bonds between its own molecules.^83^

The contact angle measurements provide valuable insights into the surface characteristics of chitosan matrices, emphasizing the influence of molecular weight on wettability and surface energy.^84^ Wettability is a key surface parameter for topical drug delivery systems because it can directly influence the interaction with the skin surface. It may be assumed that more hydrophilic surfaces are particularly beneficial for topical applications because they improve initial skin wetting, enhance intimate contact with the stratum corneum, and may promote better adhesion to the skin surface. In this study, all tested chitosan matrices exhibited hydrophilic properties, as indicated by the relatively low water contact angles. Notably, the 2CS_M and 2CS_M/IBU samples demonstrated significantly lower water contact angles and higher surface free energy compared to the other formulations, suggesting a superior ability to interact with water. The observed trends align with the molecular weight of the chitosan used. Matrices constructed from medium molecular weight chitosan (2CS_M and 2CS_M/IBU) showed significantly lower diiodomethane contact angles than those made from low molecular weight chitosan, indicating a more hydrophilic surface character governed by the polymer itself. After IBU loading, the wettability of 2CS_M remained comparable to that of native chitosan, suggesting that the outermost interface is still dominated by chitosan-derived –OH and –NH groups, rather than by the hydrophobic aromatic moieties of IBU.^85^ Furthermore, partial deprotonation of ibuprofen (carboxylate formation) during NaOH neutralization may promote hydrogen bonding and ionic interactions with the polymer, limiting the contribution of hydrophobic fragments at the surface.^86,87^ The lack of a clear correlation between AFM-derived roughness and wettability is consistent with earlier reports showing that surface chemistry outweighs topographical effects in hydrophilic polymer systems.

This correlation suggests that higher molecular weight chitosan offers enhanced wettability, likely due to greater chain flexibility and hydrophilic functional groups available for interaction with water and other liquids.^88^ Although IBU is a hydrophobic drug, its incorporation into CS matrices does not necessarily result in a pronounced decrease in surface wettability when polar functionalities dominate the interface. Previous studies have shown that the surface exposure of hydrophilic groups inherent to polysaccharide chains can control macroscopic wettability, even in the presence of hydrophobic drug molecules.^89^ This suggests that the IBU addition does not compromise the hydrophilicity imparted by the CS itself. This finding could have implications for the development of drug delivery systems, where maintaining surface properties is crucial for effective release and interaction with biological systems. Wettability is one of the key parameters in biomaterial surface evaluation. This parameter affects biological response and effectiveness, such as protein, cell, and bacterial adsorption, platelet adhesion and/or activation, and blood coagulation.^90,91^ Comparable wettability ranges have been reported for other chitosan-based biomedical systems. Dos Santos *et al.* reported water contact angles of 64.2° in distilled water (pH 5.2) and 67.7° in PBS (pH 7.2) for neat chitosan films designed for wound-dressing applications.^92^ After incorporation of hydrophobic essential oils, the contact angles decreased to 53.7° and 47.7° in water and to 62.8°, 58.3°, and 56.9° in PBS, depending on the additive, while remaining within the hydrophilic range relevant for skin contact. Similarly, Dragar *et al.* showed that loading hydrophilic polymer nanofibrous mats composed of polyethylene oxide and poloxamer with ibuprofen caused only marginal changes in wettability, with contact angles of 53.8 ± 8.8° for drug-free mats and 51.8 ± 8.0° after IBU incorporation.^93^ In addition, Peng *et al.* reported a water contact angle of 82.23° for optimized carboxymethyl chitosan/gelatin films containing *Litsea cubeba* essential oil nanoemulsions stabilized by whey protein for wound-healing applications, confirming that moderately hydrophilic surfaces are commonly reported for chitosan-based biomaterials.^94^ These observations therefore indicate that comparable wettability ranges have been reported for other chitosan-based biomedical systems. The wettability values obtained in this study are consistent with those reported for comparable chitosan-based biomedical systems, where moderately hydrophilic surfaces (water contact angles typically in the range of ∼50–80°) are considered favorable for skin-contact and drug-delivery applications and are only marginally affected by the incorporation of hydrophobic additives such as ibuprofen.^92–94^

These findings are crucial for understanding the behavior of chitosan–ibuprofen systems, optimizing drug delivery applications, and tailoring surface properties for specific biomedical uses. These applications are mainly attributed to CS structure rich in functional amino and hydroxyl moieties, which enable its modifications in a facile manner, as well as effectively binding active substances, like proteins or polynucleotides, for the precisely dosed and sustained release of bioactive substances or drugs.^95^ Further studies could explore the long-term stability of these conjugates and their implications for the functional performance of the materials.

## 5. Conclusions

The combined ATR FT-IR, AFM, and wettability analyses indicate that ibuprofen is uniformly incorporated into CS matrices without aggregation, with different binding mechanisms based on MW. Spectral data reveal changes in molecular structure, while AFM analysis shows variations in surface roughness and grain size, but homogeneous distribution confirms the successful incorporation and release of ibuprofen. Wettability tests further demonstrate that MMW CS matrices (2CS_M and 2CS_M/IBU) have significantly lower water and diiodomethane contact angles and higher SFE compared to LMW CS samples, indicating their superior wettability. The addition of ibuprofen did not affect the wetting properties of the CS films. Importantly, among all tested samples only the matrix based on MMW CS showed sustained and prolonged release of the ibuprofen, which is beneficial in the case of topical application on the skin. Considering wettability test results, it may be assumed that the hydrophilicity of the sample and its SFE directly affects the drug release profile.

## Conflicts of interest

There are no conflicts to declare.

## Supplementary Material

RA-016-D6RA00296J-s001

## Data Availability

Data for this article, including spectroscopic data, are available at Zenodo at https://doi.org/10.5281/zenodo.18183683. Supplementary information (SI) is available. See DOI: https://doi.org/10.1039/d6ra00296j.
