# Supplementary material for: Molecular and physicochemical arrangement of chitosan–ibuprofen matrices for topical drug delivery on skin: preparation impact
Source: RSC Adv. 2026 Mar 19;16(17):15591–608. doi: 10.1039/d6ra00296j (PMC13001597; doi:10.1039/d6ra00296j)
Supplement: RA-016-D6RA00296J-s001 [file RA-016-D6RA00296J-s001.pdf]

Supplementary Information (SI) for RSC Advances.
This journal is © The Royal Society of Chemistry 2026

Supplementary

Table S1. The most important bands recorded in the ATR FT-IR spectra of chitosan matrices and assigned particular components.

Wavenumber [cm^{-1}]	Assignment
3355	N-H (stretch.)
3280	−CONH−, OH (stretch.)
2920	CH_2, CH_3 (stretch.)
2872	C-H (stretch.)
1645	C=O (Amide I)
1556	C=O (Amide II)
1420	CH_2 (bending)
1408	C-H (def. sym.)
1375	C-N (stretch.)
1314	C-H (def. in-plane)
1254	C-H (def. in-plane)
1150	C-O (stretch.)
1056	C-O-C (ring)
1024	C-O (stretch.)
990	C-H (def. out-of-plane)
945	C-H (def. out-of-plane)
893	C-H (def. out-of-plane)
661	COO- (asym. stretch.)
556	C-C-C (def.)
514	C-N-C (bending)

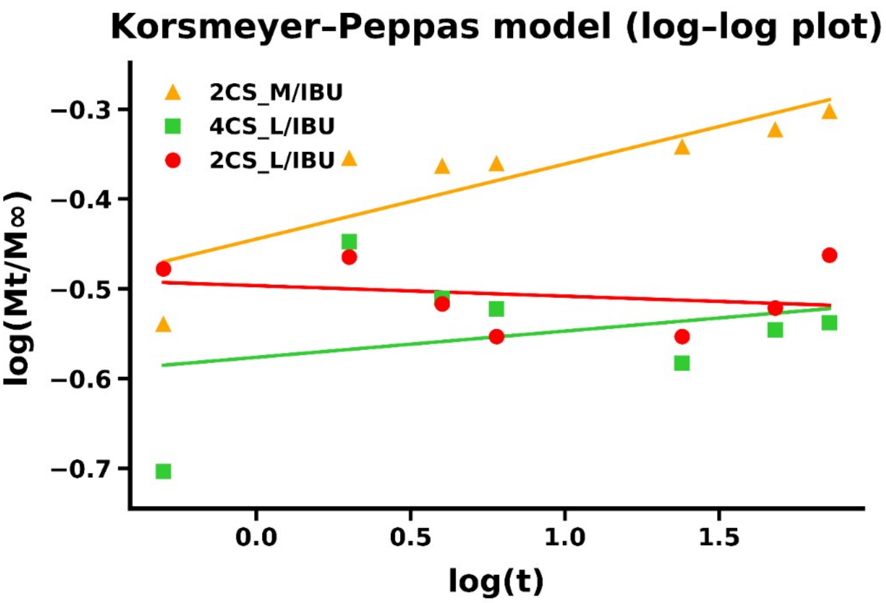

Fig. S1. Korsmeyer–Peppas log–log plot of ibuprofen release from 2CS_L/IBU, 4CS_L/IBU, and 2CS_M/IBU matrices (M_t/M_∞ < 0.6) with linear regression lines used to determine kinetic parameters (*k*, *n*) and correlation coefficients (R^2).

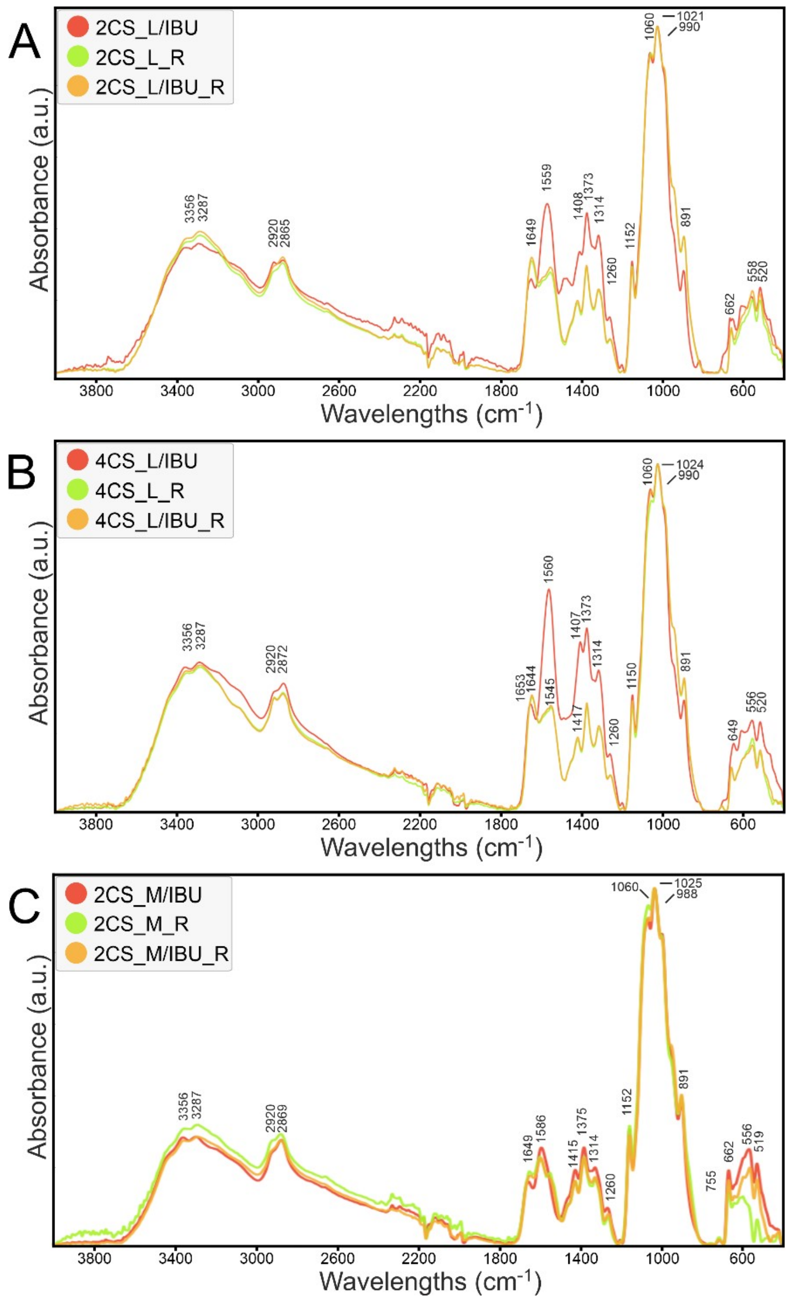

Fig. S2. ATR FT-IR spectra of 2CS_L (A), 4CS_L (B), and 2CS_M (C) with and without ibuprofen normalized to the highest intensity band at ~1021 cm^{-1}.

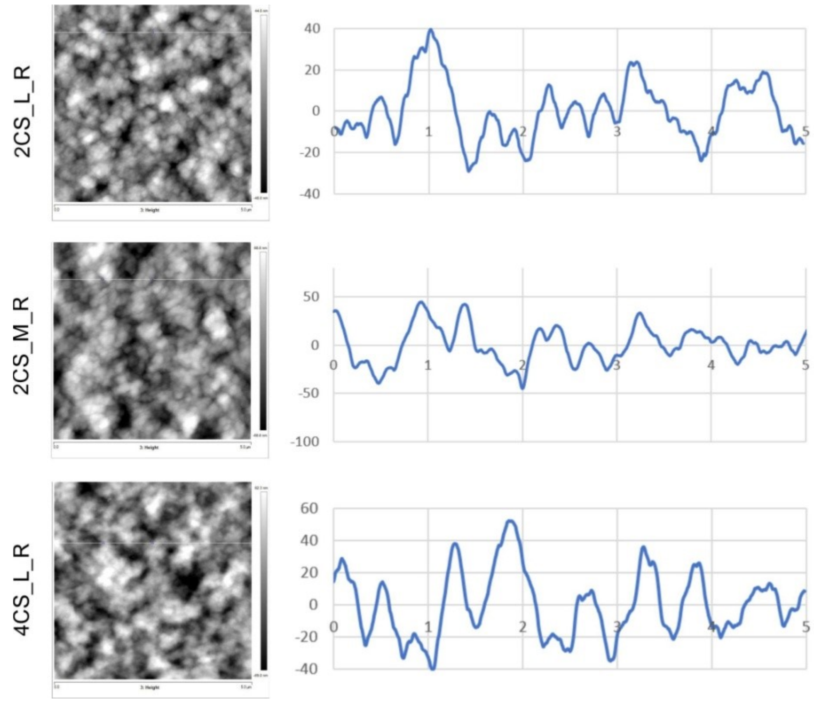

Figure S3. Linear profiles of 2CS_L_R, 2CS_M_R and 4CS_L_R surface.

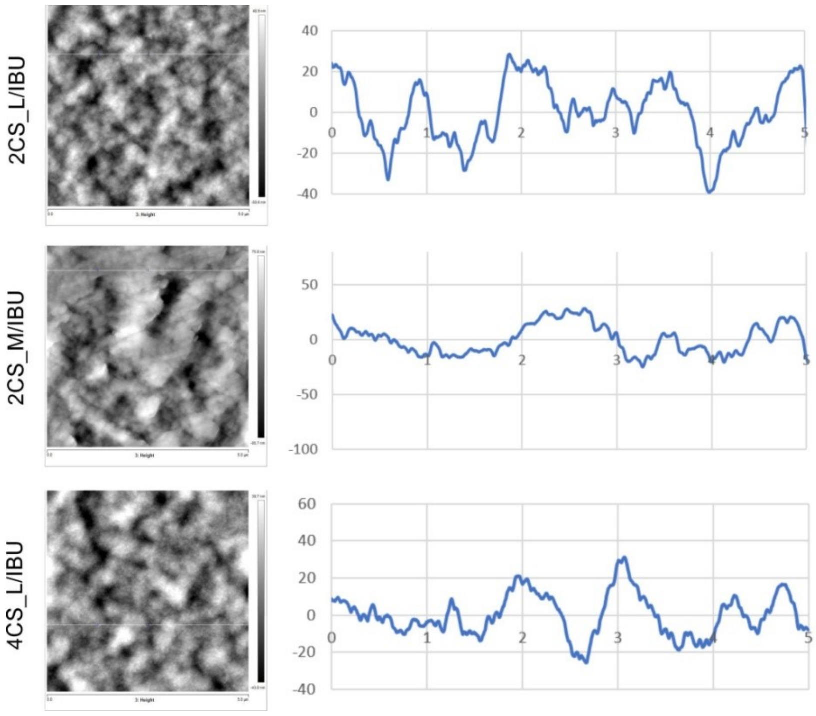

Figure S4. Linear profiles of 2CS_L/IBU, 2CS_M/IBU and 4CS_L/IBU surface.

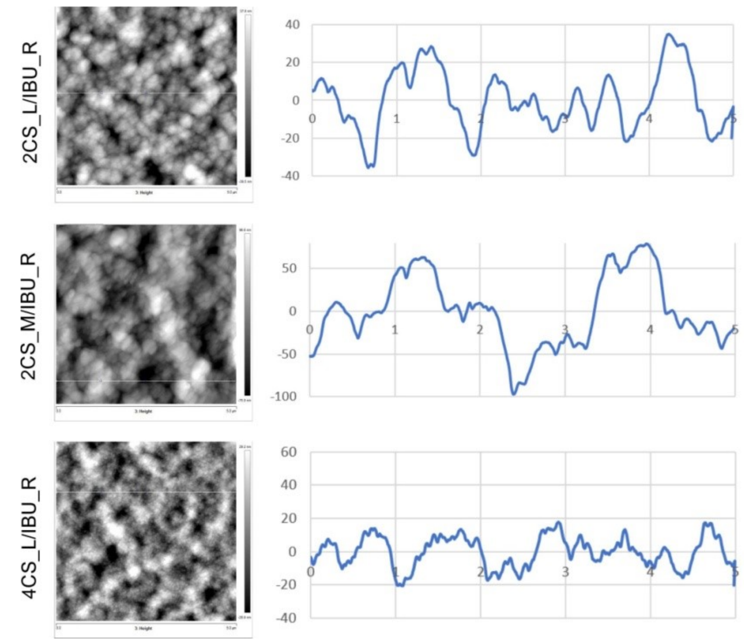

Figure S5. Linear profiles of 2CS_L/IBU_R, 2CS_M/IBU_R and 4CS_L/IBU_R surface.

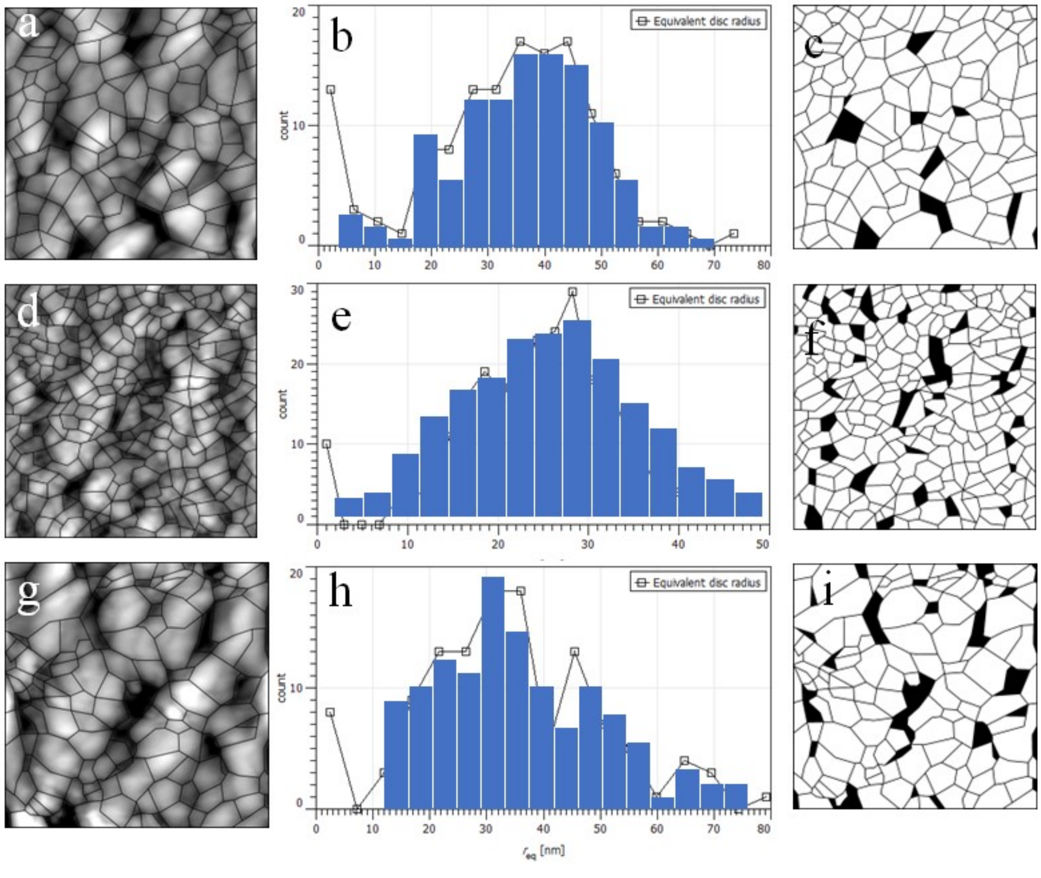

Fig. S6. AFM images of 2CS_L_R (a,c), 2CS_L/IBU (d, f), 2CS_L/IBU_R (g, i) and grain size distribution histograms (b, e, h) with specified number of grains analyzed (c, f, i); zoom in are 800 nm x 800 nm.
